# Microbial activity of lactic acid bacteria and hydrogen producers mediated by pH and total solids during the consolidated bioprocessing of agave bagasse

**DOI:** 10.1007/s11274-024-03888-1

**Published:** 2024-01-16

**Authors:** Karol Dudek, Cecilia Lizeth Álvarez Guzmán, Idania Valdez-Vazquez

**Affiliations:** Instituto de Ingeniería, Unidad Académica Juriquilla, Universidad Nacional Autónoma de México, Blvd. Juriquilla 3001, Querétaro, 76230 Mexico

**Keywords:** Consolidated bioprocess, *Clostridium sensu stricto* 1, Lactic acid bacteria, Lignocellulosic biomass, *Enterococcus*

## Abstract

Lactic acid bacteria (LAB) coexist with *Clostridium* spp. in hydrogen production processes from complex substrates; however, the role of LAB is still unclear. This study analyzed the fermentation products in a wide range of initial pH (pH_i_, 5.5–6.9) and total solids (TS%, 8–22%) to determine the activity of these two microbial groups over time (from 24 to 120 h). Agave bagasse served as the feedstock for hydrogen production via consolidated bioprocess (CBP), while the inoculum source was the indigenous mature microbiota. In the early stage of the CBP, hydrogen production from lactic acid occurred only at pH_i_ ≥ 6.0 (ρ = 0.0004) with no effect of TS%; lactic acid accumulated below this pH_i_ value. In this stage, lactic acid production positively correlated with a first cluster of LAB represented by *Paucilactobacillus* (*r* = 0.64) and *Bacillus* (*r* = 0.81). After 72 h, hydrogen production positively correlated with a second group of LAB led by *Enterococcus* (*r* = 0.71) together with the hydrogen producer *Clostridium sensu stricto* 1 (*r* = 0.8) and the acetogen *Syntrophococcus* (*r* = 0.52) with the influence of TS% (ρ < 0.0001). A further experiment showed that buffering the pH to 6.5 increased and lengthened the lactic acid production, doubling the hydrogen production from 20 to 41 mL H_2_/gTS_added_. This study confirmed the prevalence of distinct groups of LAB over time, whose microbial activity promoted different routes of hydrogen production.

**Supplementary Information:**

The online version contains supplementary material available at 10.1007/s11274-024-03888-1.

## Introduction

Using lignocellulosic biomass for hydrogen production has become one of the most important lines of research in the global transition from fossil fuels to renewable resources. Moreover, lignocellulosic biomass is an attractive carbon source because it does not compete with the food industry, and its annual production is estimated at 181.5 billion tons per year (Dahmen et al. 2019). Among many types of lignocellulosic biomass, agave bagasse from the Tequila and Mezcal industries is one of the most abundant in Mexican territory. Its annual dry weight is estimated at 149,777 tons (Hernández et al. 2019). The biomass composition ranges from 38 to 53% cellulose, 32–54% hemicellulose, 4–14% lignin, and up to 20% extractives. The compositional variability is conditioned by climate, cultivation, agronomic practices, harvesting time, and plant age. Its biodegradability is low and is affected by several factors, such as the recalcitrant character of the lignocellulosic biomass, polymeric and crystalline degree of cellulose, and the branching nature of hemicelluloses and lignin polymers. Therefore, physicochemical or biological pretreatment is required to break down the complex structure of hemicellulose and cellulose into pentoses (mainly xylose) and glucose, respectively (Dudek et al. 2021).

Alternatively, a consolidated bioprocess (CBP) could be applied due to its advantage of combining three processes occurring in the same reactor: production of saccharolytic enzymes, hydrolysis of the polysaccharides, and pentose and hexose fermentation (Dudek et al. 2021). Indigenous microbiota in lignocelluloses are a promising biocatalyst to perform the CBP for hydrogen production (Ayala-Campos et al. 2022). Recent research has evidenced how different indigenous microbiota converged into a core microbiome formed by *Clostridium* and *Lactobacillus*, genera that were positively associated with hydrogen production. In the literature, there is still no consensus about the role of lactic acid bacteria in hydrogen production processes (Castelló et al. 2020). However, an increasing number of studies have shown the feasibility of producing hydrogen from lactic/acetic acids (García-Depraect et al. 2021). LAB comprise diverse genera, some of which have been reported in hydrogen production processes via CBP. The genus *Enterococcus* was probably the first lactic acid bacteria reported in reactors that produced hydrogen via CBP from untreated wheat straw thanks to their xylanolytic activity (Valdez-Vazquez et al. 2015, 2017). On the other hand, the lactic acid bacterium *Lactobacillus* has also been reported in hydrogen-producing reactors via CBP (Ayala-Campos et al. 2022; Pérez-Rangel et al. 2023). These two LAB seem to establish different interactions with hydrogen producers; for example, Pérez-Rangel et al. (2022) reported antagonism between *Lactobacillus* – *Enterococcus  and Enterococcus* – *Clostridium*, but not between *Lactobacillus* –*Clostridium*. Notably, in those processes where *Lactobacillus* and *Clostridium* thieved together, there was no accumulation of lactic acid, which strongly suggests the occurrence of the lactic acid-based pathway.

This study aims to determine the temporal prevalence of LAB and hydrogen producers during the consolidated bioprocess of agave bagasse. To this end, the reactors were loaded with different contents of total solids and initial pHs because these two factors regulate the metabolic pathways established during fermentation of complex substrates (Motte et al. 2013; Sarkar et al. 2021), offering the opportunity to capture different behaviors. Once the best performance was identified, the consolidated bioprocess was monitored every 24 h until the hydrogen production stabilized.

## Materials and methods

### Substrate and inoculum

The agave bagasse used for the experiments was collected from Tequilera Real de Penjamo in Penjamo, Guanajuato, Mexico. The biomass was sun-dried and stored in a closed plastic container at room temperature. Before its use, the lignocellulosic biomass was milled using an industrial mixer (LI-3 A, VECA INTERNATIONAL), and particles between 2 and 4 mm were selected using sieves (Endecotts, London). The agave bagasse composition was as follows (in dry matter): extractives 16%, cellulose 41%, hemicellulose 22%, lignin 16%, ash 4% (Hernández et al. 2019; Ayala-Campos et al. 2022).

The native microbiota of nonsterile agave bagasse at 80% moisture content was used to inoculate the reactors (Dudek et al. 2021), which were operated for eight weeks under the following conditions: TS 15%, initial pH of 6.5, which was adjusted at each feeding, 37℃, and agitation of 150 rpm, operational conditions previously selected for producing hydrogen from untreated lignocellulosic feedstocks (Ayala-Campos et al. 2022; Pérez-Rangel et al. 2023). The culture medium contained (g/L): 1.02 of urea, 0.41 CaCl_2_, and 0.11 KH_2_PO_4,_ according to Pérez-Rangel et al. (2020).

### Experimental design

The aim of this study was to investigate the prevalence of LAB and hydrogen producers at different levels of pH_i_ and TS. To buffer pH_i_, the 2-(N-morpholino)ethane-sulfonic acid (MES) was chosen; in consequence, the experimental pH ranged from 5.5 to 6.9. The levels of the TS content to be studied were selected based on a literature review (Yang et al. 2015; Sun et al. 2021). The central composite design (CCD), which consisted of two numeric factors at two levels: initial pH at 5.7 (low level), 6.2 (center point), and 6.7 (high level), and TS at 10% (low level), 15% (center point), and 20% (high level), was used to study the microbial activity. In this way, 13 runs were obtained, where eight runs represented the non-center points and five runs the center points (or replicates).

A quadratic model (Eq. 1) was used to assess the relationship between the response variable (VFA concentration) and the factors (*X*_*1*_—pH, *X*_*2*_—TS) based on experimental data.

1 $${Y}_{i}= {\beta }_{0}+ \sum {\beta }_{i}{X}_{i}+\sum {\beta }_{ii}{X}_{i}^{2}+ \sum {\beta }_{ij}{X}_{i}{X}_{j}$$

Where ${Y}_{i}$ is the response variable and expresses VFA concentration in g/L, ${\beta }_{0}$ is the constant, ${\beta }_{i}$ is the linear coefficient, ${\beta }_{ii}$ is the squared coefficient, and ${\beta }_{ij}$ is the interaction coefficient. The experimental design, statistical analysis, and construction of 3D response surface plots were prepared using Design Expert v10 (Stat-Ease, Inc., MN, USA).

The experiments were conducted in 250 mL glass flask bottles (Bellco Glass, Shrewsbury, UK) with a working volume of 100 mL. Into each reactor, 10 g of inoculum (85% of moisture) was introduced. The culture medium, TS%, and initial pH varied according to the experimental design. The culture medium contained 100 mM MES buffer and the following nutrients (g/L): 1.02 of CH_4_N_2_O, 0.41 CaCl_2_, and 0.11 KH_2_PO_4_ (Pérez-Rangel et al. 2020). The reactors were sealed tightly and incubated at 37 °C, with the agitation of 150 rpm for five days. Every 24 h a fermentation broth sample was taken while the gases were released into the atmosphere.

### Validation experiment

The experimental condition that led to the highest concentration of butyric acid was chosen as the optimal condition and validated in an additional set of experiments. The run was as follows: initial pH 6.5, TS 22.1%, with the above-mentioned culture medium composition. Two treatments were applied: reactors with a low buffer capacity using 100 mM MES and reactors with pH buffering to 6.6 using a NaOH solution. For each buffer capacity, 15 identical reactors with pH_i_ 6.5 and TS 22.1% were prepared. For all reactors, every 12 h, the gases were measured and then released into the atmosphere. Also, at times 0, 24, 36, 48, and 72 h, three reactors were discarded from the experiment to take liquid and solid samples for further analytical and molecular analyses.

### Analytical methods

The pH was measured using a potentiometer (Beckman, 50 pH Meter). Concentrations of volatile fatty acids (VFAs) were analyzed using high-performance liquid chromatography (HPLC) with 10 µL sample injection (model 1260 infinity, Agilent Technologies, CA, USA) equipped with an Aminex HPX-87 H column and two detectors: Refractive Index Detector (RID) and Diode-Array Detector (DAD) with a detection wavelength of 210 nm. The mobile phase was a 5 mM H_2_SO_4_ solution at a 0.6 mL/min flow rate. Biogas composition (H_2_, CH_4_, and CO_2_) was analyzed with gas chromatography (GC) (SRI Instruments Model 8610 C, Champaign, IL, USA) equipped with a thermal conductivity detector (TCD) and two steel columns (2 m in length; 0.79 mm in diameter). The injector, column, and detector temperatures were 90, 110, and 150 °C, respectively. Nitrogen was used as a carrier gas at a 20 mL/min flow rate. Gas volume was reported at standard temperature and pressure (0 °C and 1013.25 hPa).

### DNA extraction and molecular analyses

Triplicate samples at 0, 24, 36, 48, and 72 h were stored at – 80 ºC until processing. DNA extraction was performed for each sample by using the PowerSoil DNA extraction kit® (MoBio Laboratories Inc., Carlsbad, CA, USA) following the manufacturer’s instructions. The quality of the extracted DNA was verified by using the A260/280 and A260/230 ratios over to 1.8 and 2.0, respectively. Then, the DNA concentrations were adjusted to 20 µg/µL by using DNase/Pyrogen-free water. Processed samples were sequenced individually by using the Illumina MiSeq platform with the primer set 28 F (GAGTTTGATCNTGGCTCAG) and 388R (TGCTGCCTCCCGTAGGAGT). Raw sequences were processed following the pipeline previously described (Pérez- Rangel et al., 2021).

### Statistical analysis

The experimental design, statistical analysis, and construction of 3D response surface plots were prepared by using Design Expert v10 (Stat-Ease, Inc., MN, USA). Canonical correlation analysis was used to identify and measure the associations between fermentation products, time, and microbial community by using Past 4.11 (Hammer et al. 2001).

## Results

### Fermentation performance at different pH_i_ and TS%

**Fig. 1:**
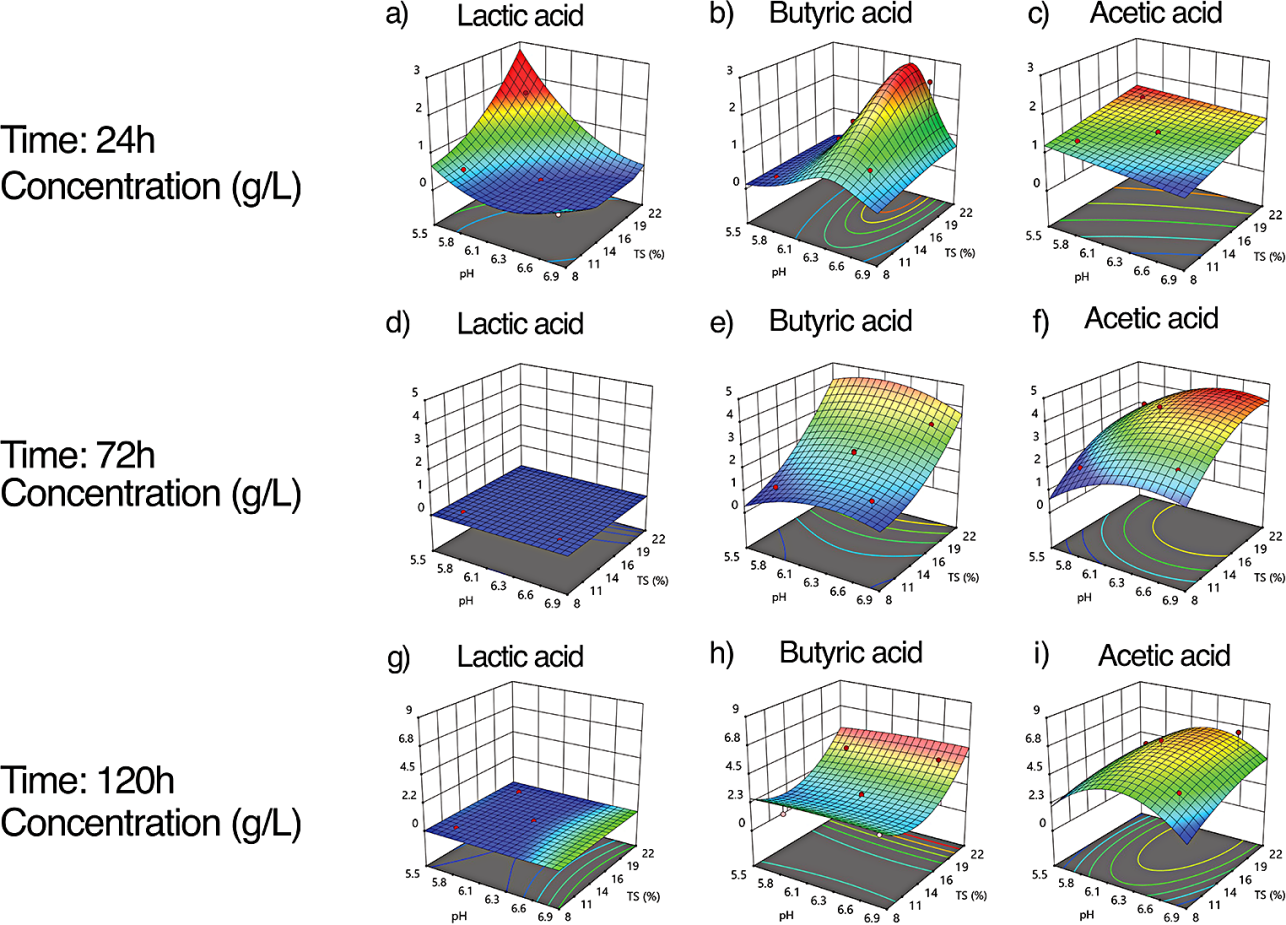
Effects of TS% and initial pH (pH_i_) on the production of the lactic, butyric, and acetic acids after 24, 72, and 120 h during CBP of agave bagasse

Lactic acid production was observed only at the beginning of the fermentation (24 h) (Fig. 1a). The ANOVA indicated that pH_i_ (ρ = 0.0003) and TS content (ρ = 0.0200) had a significant effect on lactic acid production: the highest experimental concentration reached was 2.8 g/L at pHi 5.7 and TS of 20%. Lactic acid production increased when pH decreased and TS% increased. As long as the pH_i_ was higher than 6.0, lactic acid production was not visible, regardless of the TS content (Figure S1 displays lactic acid production as g/g_TS_). The numerical optimization predicted the maximum lactic acid concentration (${C}_{HLa}$) of 1.05 g/L at the pH_i_ 5.86 and TS content of 20%. The experimental lactic acid production had a ${R}^{2}$ adjusted in 83.5% to its predicted production by the model.

2 $$\begin{aligned} {Ln (C}_{HLa}+0.0017)& =-6.09-2.71{X}_{1}+1.26 {X}_{2} \\ & \quad -0.3158{X}_{1}{X}_{2}+1.48{{X}_{1}}^{2}+1.42{{X}_{2}}^{2} \end{aligned}$$

Butyric acid was found in the fermentation broth after 24 h (Fig. 1b). The ANOVA pointed out that only pH_i_ (ρ = 0.0004) significantly affected butyric acid production, while the TS% did not (ρ = 0.0576). Despite the statistical analysis, the upward trend in butyric acid production was observed at a TS% increase. The butyric acid production was observed for the pH_i_ between 6.0 and 6.9 (Figure S1 displays butyric acid production as g/g_TS_). The numerical optimization predicted the maximum butyric acid concentration ($${C}_{HBa})$$of 4.0 g/L at the pH_i_ 6.39 and TS content of 20% with desirability of 0.824. The experimental butyric acid production had a $${R}^{2}$$ adjusted in 88.8%.

3 $$\begin{aligned} {\frac{1}{Sqrt} (C}_{HBa})& =+89.76-27.45{X}_{1}-0.13 {X}_{2}\\ & \quad -0.01 {X}_{1}{X}_{2}+2.14{{X}_{1}}^{2}+0.002{{X}_{2}}^{2} \end{aligned}$$

Acetic acid was the third most abundant metabolite detected in the reactors after 24 h (Fig. 1c). The ANOVA showed that pH_i_ (ρ = 0.0334) and TS% (ρ = 0.0008) significantly impacted acetic acid formation. Its maximum experimental concentration of 1.6 g/L was found for pH_i_ 5.7 and TS 20%. Its concentration increased with a decrease in pH_i_ and an increase in TS content (Figure S1 displays acetic acid production as g/g_TS_). The numerical optimization predicted the maximum acetic acid concentration (${C}_{HAc}$) of 1.6 g/L at the pH_i_ 5.7 and TS content of 20% with desirability of 0.918. The experimental butyric acid production had a ${R}^{2}$ adjusted in 70.3%.

4 $${C}_{HLa}=1.30-0.0984{X}_{1}+0.1961 {X}_{2}+0.0231{X}_{1}{X}_{2}$$

After 72 h of fermentation, lactic acid was undetected (Fig. 1d). Butyric acid reached its maximum concentration of 4.8 g/L for pH_i_ and TS 22% (Fig. 1e). The statistical analysis of ANOVA indicated that only TS% (ρ < 0.0001) was significant, but not pH_i_ (ρ = 0.8501). Butyric acid concentration increased with increasing TS%, while changes in its concentration were not observed at different pH values. The maximum ${C}_{HBa}$of 3.78 g/L was predicted at pH_i_ 6.1 and TS 20%, desirability of 0.748. The experimental butyric acid production had a ${R}^{2}$ adjusted in 93.7%.

5 $$\begin{aligned} {C}_{HBa}& =-52.62+16.61{X}_{1}+0.19 {X}_{2} \\ & \quad -0.06 {X}_{1}{X}_{2}-1.27{{X}_{1}}^{2}+0.001{{X}_{2}}^{2} \end{aligned}$$

Acetic acid production after 72 h resulted in significant dependence on pH_i_ (ρ < 0.0001) and TS% (ρ < 0.0001). Its maximum experimental concentration of 4.3 g/L was detected at pH_i_ 6.2 and TS 15%. Acetic acid production was more influenced by an increase in TS% then the pH_i_ more alkaline (Fig. 1f). The maximum ${C}_{HAc}$of 4.5 g/L was predicted at the pH_i_ 6.54 and TS content of 20% with desirability of 0.998. The experimental butyric acid production had a ${R}^{2}$ adjusted in 94.5%.

6 $$\begin{aligned} {C}_{HBa}& =3.88+0.5219 {X}_{1}+0.7883 {X}_{2} \\ & \quad +0.0383 {X}_{1}{X}_{2}-0.4138 {{X}_{1}}^{2}-0.3648{{X}_{2}}^{2} \end{aligned}$$

After 120 h there was no lactic acid at all (Fig. 1g). Butyric acid production (Fig. 1h) had a concentration similar to that one reached after 72 h. However, its maximum concentration was shifted to pH_i_ 6.6. The statistical analysis of ANOVA indicated that only TS% (ρ = 0.0069) was significant, but not pH_i_ (ρ = 0.6796). The maximum ${C}_{HBa}$ of 4.06 g/L was predicted at pH_i_ 6.66 and TS 20%, desirability of 0.796. The experimental butyric acid production had a ${R}^{2}$ adjusted in 68.7%.

7 $$\begin{aligned} {C}_{HBa}& =-3.47+3.96 {X}_{1}-1.16 {X}_{2} \\ & \quad +0.02 {X}_{1}{X}_{2}-0.33 {{X}_{1}}^{2}+0.04{{X}_{2}}^{2} \end{aligned}$$

At 120 h, acetic acid peaked at pH_i_ 6.2 and TS 15% with a concentration of 6.7 g/L. The quadratic model applied to the collected data was not significant at that time. Finally, propionic acid was found at its highest concentration of 2.3 g/L at pH_i_ 6.7 and TS 20% (data not shown). The response surface model was neither significant at 120 h, nor at other fermentation times of the kinetic. This suggests that other factors, not considered in this study, affected its production.

### Validation experiment

**Fig. 2:**
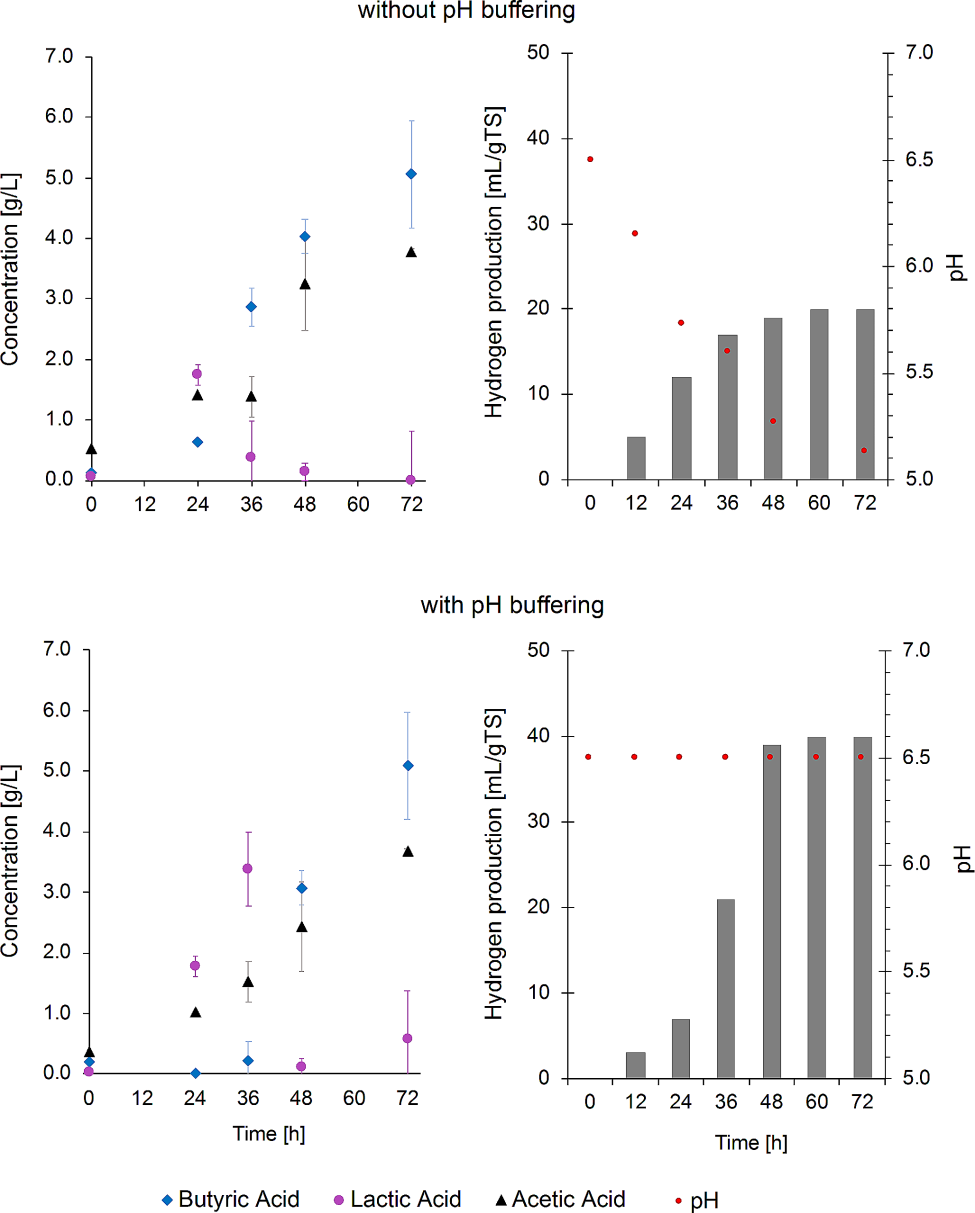
Influence of the pH buffering on the stability and fermentation products from agave bagasse via CBP

The buffer capacity influenced the LAB activity and, consequently, the hydrogen production (Fig. 2). The reactor with pH buffering to 6.5 yielded the maximum concentration of lactic acid of 3.4 g/L after 36 h. In contrast, lactic acid production peaked at 24 h at 1.7 g/L in the reactor without pH buffering. The hydrogen production doubled in the reactors with pH buffering at the expense of the consumption of lactic acid.

### Correlation between fermentation products and microbial diversity

**Fig. 3:**
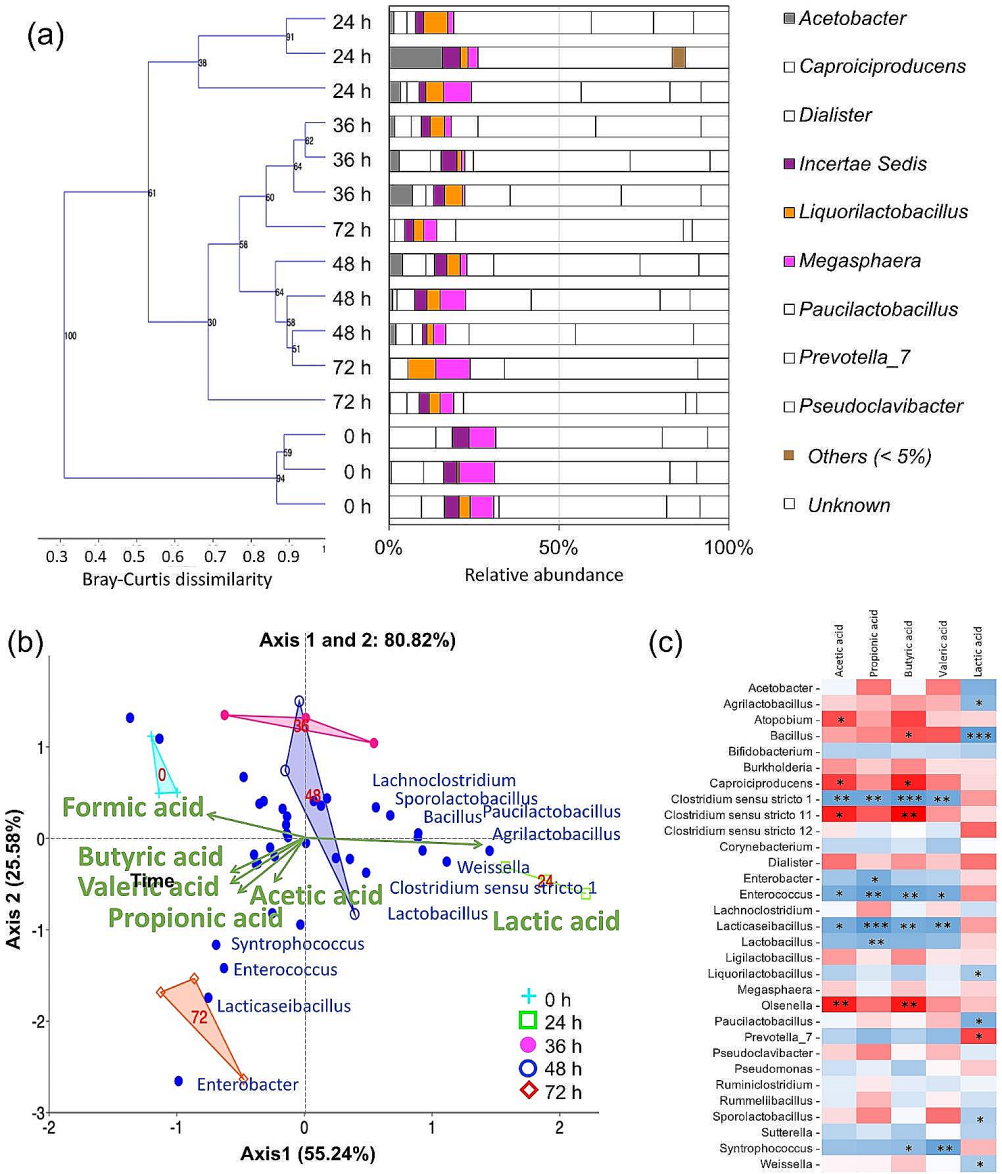
**a** Dendrogram based on Bray-Curtis distances obtained by UPGAMA clustering and the relative abundances of bacterial genera (> 5%) during the consolidated bioprocessing of agave bagasse for hydrogen production. **b** Canonical correspondence analysis (CCA) triplot (*n* = 15, three per day) of species, fermentation time, and products. **c** Pearson correlations between bacterial genera and products. Blue color (positive r values) indicates likelihood of co-occurrence and red color (negative r values) indicates no relationship, not necessarily a negative correlation. Stars inside each cell represent the significance of the correlation (*** = 0.001; ** = 0.01; * = 0.05)

Bacterial diversity was analyzed at times 0, 24, 36, 48, and 72 h of CBP of agave bagasse with pH_i_ 6.5 and TS 22.1% (validation experiment, reactor without pH buffering). Bacterial communities clustered by time being 0 and 24 h two distant groups (Fig. 3a). Bacterial communities present during the times between 36, 48 and 72 h clustered together. The *Prevotella* genus was ubiquitous and abundant during the experiment. This genus has been previously identified in mature fermentation of agave bagasse and is linked to the degradation of polysaccharides such as xylan (Dudek et al., 2020). A canonical correspondence analysis (CCA) of species, fermentation time, and products showed that *Bacillus*, *Agrilactobacillus*, *Sporolactobacillus*, *Paucilactobacillus*, and *Weissella*—present at 24 h—correlated with the lactic acid production (Fig. 3b,c). Bacteria did not correlate with fermentation products at times 36 and 48 h. Finally, at 72 h, *Clostridium senso stricto* 1, *Enterococcus*, *Lacticaseinacillus*, and *Syntrophococcus* correlated with most fermentation products (Fig. 3b,c).

## Discussion

Fermentation products were presented as final concentration (Fig. 1) and as absolute values in relation to the TS added (Figure S1). For the butyric acid production at 24 h, both graphs showed that pH_i_ significantly influenced the microbial activity, where the maximum values occurred at pHi ≥ 6.3, whereas TS% did not influence. The remaining response variables showed the same trends in both graphs. The analysis of fermentation products over time evidenced different fermentation behaviors at 24 and 72 h depending on the pH_i_ and TS%; beyond this time, these two operational parameters did not influence the CBP. At 24 h, the lactic acid and butyric acid occurred at opposite extremes of pH_i_ and TS% of 20; the lactic acid production peaked at pH_i_ 5.5 (2.8 g/L) while the butyric acid production peaked at pH_i_ 6.39. A further kinetic analysis evidenced that lactic acid disappeared as soon as the hydrogen production started. Lactic acid decomposition to hydrogen and butyric acid occurs through two major pathways of biochemical metabolism: the acrylate pathway and the pyruvate-ferredoxin oxidoreductase pathway carried out by *Clostridium* spp. (Tholozan et al. 1992). According to García-Depraect et al. (2021), mentioned pathways take place at pH values between 5.5 and 6.0, this pH range agreed with the results of this study, since no hydrogen production was observed at pHs below 5.5. At this time, an early-stage microbial cluster of LAB identified as *Agrilactobacillus*, *Liquorilactobacillus*, *Paucilactobacillus*, *Sporolactobacillus*, *Weissella*, and *Bacillus* positively correlated with lactic acid production, but no with hydrogen production.

By 72 h, the pH_i_ dropped at least 1.0 unit in all reactors; under such conditions, lactic acid was undetected, and the hydrogen/butyric acid production depended only on the levels of substrate availability. At this time, a late-stage microbial cluster of LAB identified as *Enterococcus* and *Lacticaseibacillus*, together with the hydrogen producer *Clostridium sensu stricto* 1 and the acetogen *Syntrophococcus*, positively correlated with hydrogen/butyric acid production. In this late-stage microbial cluster, *Clostridium sensu stricto* 1 and *Enterococcus* possibly produced hydrogen from the carbohydrate consumption (Luo et al. 2023; Valdez-Vazquez et al. 2015), as opposed to the lactic acid used as substrate in the early stage. *Enterococcus* is of particular interest since it has been previously identified in endpoint hydrogen-producing processes (Valdez-Vazquez et al. 2017; Ayala-Campos et al. 2022), whose isolates demonstrated xylanolytic activity on oat-spelt xylan (Valdez-Vazquez et al. 2015). On the other hand, pioneer studies also identified the acetogen *Syntrophococcus* in CBP devoted to producing hydrogen (Navarro-Díaz et al. 2016; Valdez-Vazquez et al. 2017). The *Syntrophococcus* genus is able to form a symbiosis with other anaerobes that metabolize the ligno-aromatic compounds (Bernard-Vailhe et al. 1995). The prevalence of *Syntrophococcus* at the late stage indicates its possible participation in the degradation of xylan-lignin bonds, which calls for further research.

These results indicate that the *Clostridium* spp. coexist with two groups of LAB during the CBP of agave bagasse. An early-stage microbial cluster where LAB produced lactic acid, which served as a substrate for hydrogen producers (a pH-dependent process), establishing a cross-feeding interaction with *Clostridium* spp. However, a late-stage microbial cluster was represented by *Enterococcus*, a genus that could compete with *Clostridium* spp. for carbohydrates to produce hydrogen. The optimal, stable performance of hydrogen-producing reactors relies on beneficial microbial interactions between members of a microbial consortium. Since LAB are ubiquitous in vegetable materials such as lignocellulosic feedstocks, it is necessary to determine which group of LAB is more convenient for producing hydrogen in stable, productive reactors. In this study, the pH buffering favored the lactic acid production, increasing the hydrogen yield. The hydrogen production from the lactic acid consumption seems more productive since it is based on the positive relationship between LAB and the *Clostridium* spp. Future research must determine if separating these two processes (lactic acid and hydrogen production) is feasible using lignocellulosic feedstocks and its impact on the total hydrogen production cost.

### Electronic supplementary material

Below is the link to the electronic supplementary material.

Supplementary Material 1

## Data Availability

Data generated during this study are included in this published article which sequence data are available in the National Center for Biotechnology Information database under accession numbers SAMN33619598 - SAMN33619612.

## References

[CR1] Ayala-Campos OR, Sanchez A, Rebollar EA, Valdez-Vazquez I (2022). Plant-associated microbial communities converge in fermentative hydrogen production and form a core microbiome. Int J Hydrogen Energy S036031992201727X.

[CR2] Bernard-Vailhe MA, Besle JM, Dore J (1995) Transformation of 14 C-lignin-labeled cell walls of wheat by *Syntrophococcus sucromutans*, *Eubacterium oxidoreducens*, and *Neocallimastix frontalis*. Applied and Environmental Microbiology 61(1): 379–381. 10.1128/aem.61.1.379-381.1995 10.1128/aem.61.1.379-381.1995 PMC1388337 16534916

[CR3] Castelló E, Nunes Ferraz-Junior AD, Andreani C (2020). Stability problems in the hydrogen production by dark fermentation: possible causes and solutions. Renew Sustain Energy Rev.

[CR4] Dahmen N, Lewandowski I, Zibek S, Weidtmann A (2019). Integrated lignocellulosic value chains in a growing bioeconomy: Status quo and perspectives. GCB Bioenergy.

[CR5] Dudek K, Buitrón G, Valdez-Vazquez I (2021). Nutrient influence on acidogenesis and native microbial community of Agave bagasse. Ind Crops Prod.

[CR6] García-Depraect O, Castro-Muñoz R, Muñoz R (2021). A review on the factors influencing biohydrogen production from lactate: the key to unlocking enhanced dark fermentative processes. Bioresour Technol.

[CR7] Hammer O, Harper DAT, Ryan PD (2001) PAST: Paleontological Statistics Software Package for Education and Data Analysis

[CR8] Hernández C, Escamilla-Alvarado C, Sánchez A (2019). Wheat straw, corn stover, sugarcane, and *Agave* biomasses: chemical properties, availability, and cellulosic‐bioethanol production potential in Mexico. Biofuels Bioprod Bioref.

[CR9] Luo L, Lee Mak K, Mal J (2023). Effect of zero-valent iron nanoparticles on taxonomic composition and hydrogen production from kitchen waste. Bioresour Technol.

[CR10] Motte J-C, Trably E, Escudié R (2013). Total solids content: a key parameter of metabolic pathways in dry anaerobic digestion. Biotechnol Biofuels.

[CR11] Navarro-Díaz M, Valdez-Vazquez I, Escalante AE (2016). Ecological perspectives of hydrogen fermentation by microbial consortia: what we have learned and the way forward. Int J Hydrog Energy.

[CR12] Pérez-Rangel M, Barboza-Corona JE, Buitrón G, Valdez-Vazquez I (2020). Essential nutrients for improving the direct processing of raw lignocellulosic substrates through the dark fermentation process. Bioenerg Res.

[CR13] Pérez-Rangel M, Barboza-Corona JE, Navarro-Díaz M (2021). The duo *Clostridium* and *Lactobacillus* linked to hydrogen production from a lignocellulosic substrate. Water Sci Technol.

[CR15] Pérez-Rangel M, Valdez‐Vazquez I, Martínez‐Zavala SA, Casados‐Vázquez LE, Bideshi DK, Barboza‐Corona JE (2022). Evaluation of inhibitory compounds produced by bacteria isolated from a hydrogen‐producing bioreactor during the self‐fermentation of wheat straw. J Appl Microbiol.

[CR14] Pérez-Rangel M, Barboza-Corona JE, Valdez-Vazquez I (2023). Effect of the organic loading rate and temperature on hydrogen production via consolidated bioprocessing of raw lignocellulosic substrate. Int J Hydrog Energy.

[CR16] Sarkar O, Rova U, Christakopoulos P, Matsakas L (2021). Influence of initial uncontrolled pH on acidogenic fermentation of brewery spent grains to biohydrogen and volatile fatty acids production: optimization and scale-up. Bioresour Technol.

[CR17] Sun J, Zhang L, Loh K-C (2021). Review and perspectives of enhanced volatile fatty acids production from acidogenic fermentation of lignocellulosic biomass wastes. Bioresour Bioprocess.

[CR18] Tholozan JL, Touzel JP, Samain E (1992). *Clostridium neopropionicum* sp. nov., a strict anaerobic bacterium fermenting ethanol to propionate through acrylate pathway. Arch Microbiol.

[CR20] Valdez-Vazquez I, Pérez-Rangel M, Tapia A (2015). Hydrogen and butanol production from native wheat straw by synthetic microbial consortia integrated by species of *Enterococcus* and *Clostridium*. Fuel.

[CR19] Valdez-Vazquez I, Morales AL, Escalante AE (2017). History of adaptation determines short‐term shifts in performance and community structure of hydrogen‐producing microbial communities degrading wheat straw. Microb Biotechnol.

[CR21] Yang L, Xu F, Ge X, Li Y (2015). Challenges and strategies for solid-state anaerobic digestion of lignocellulosic biomass. Renew Sustain Energy Rev.

