# Supplementary material for: Microbial activity of lactic acid bacteria and hydrogen producers mediated by pH and total solids during the consolidated bioprocessing of agave bagasse
Source: World J Microbiol Biotechnol. 2024 Jan 16;40(2):70. doi: 10.1007/s11274-024-03888-1 (PMC10789659; doi:10.1007/s11274-024-03888-1)
Supplement: Supplementary file 1 — Supplementary Material 1 [file 11274_2024_3888_MOESM1_ESM.docx]

**Microbial activity of lactic acid bacteria and hydrogen producers mediated by pH and total solids during the consolidated bioprocessing of agave bagasse**

Karol Dudek, Cecilia Lizeth Álvarez Guzmán, Idania Valdez-Vazquez

**Supplementary material**


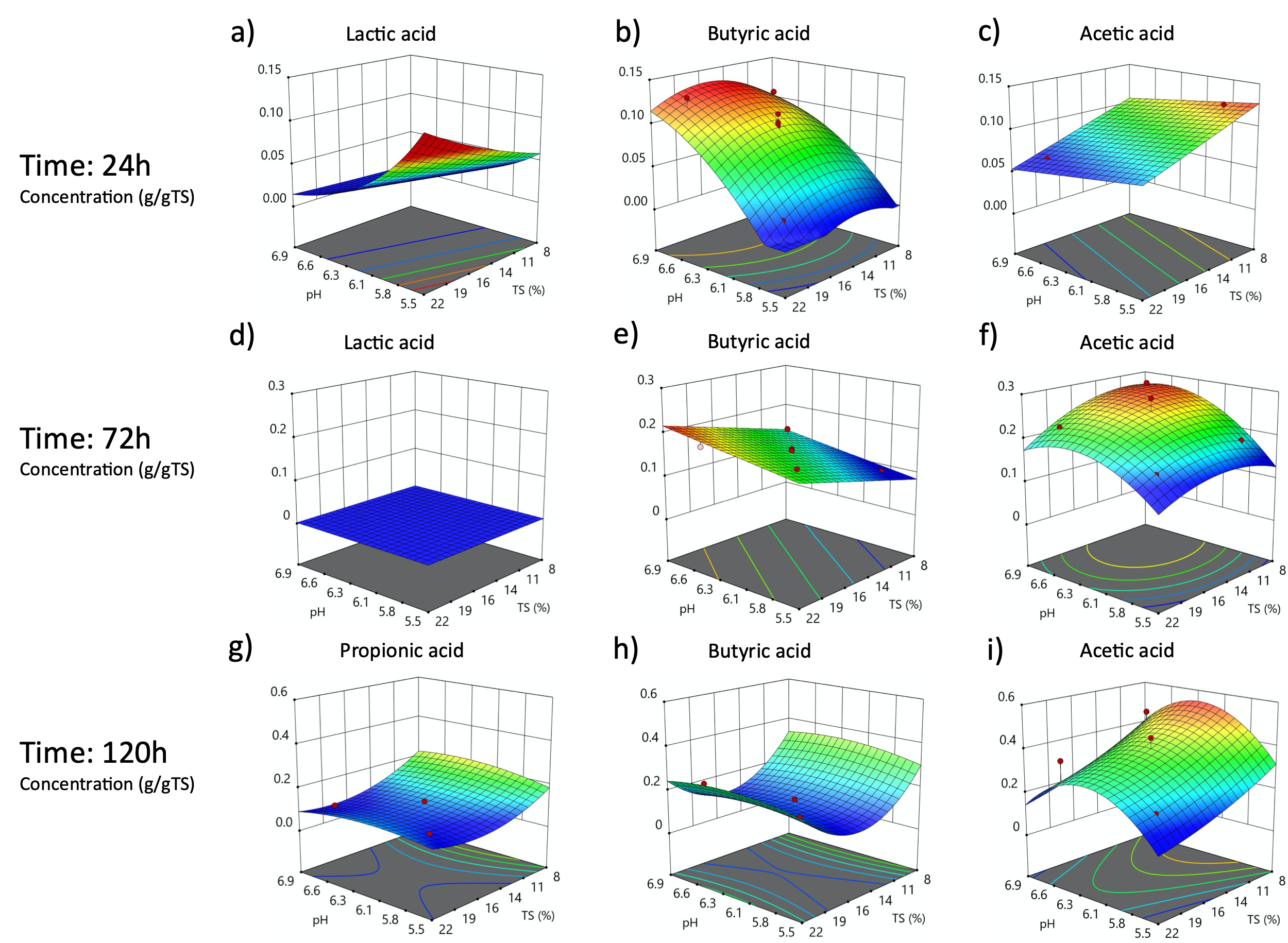


Figure S1 Effects of TS% and initial pH (pH_i_)on the production of the lactic, butyric, and acetic acids after 24, 72, and 120 h during CBP of agave bagasse (in g/g_TS_).
